# Mitochondrial DNA copy number is associated with all‐cause mortality and cardiovascular events in patients with peripheral arterial disease

**DOI:** 10.1111/joim.13027

**Published:** 2020-02-09

**Authors:** A. Koller, F. Fazzini, C. Lamina, B. Rantner, B. Kollerits, M. Stadler, P. Klein‐Weigel, G. Fraedrich, F. Kronenberg

**Affiliations:** ^1^ Department of Genetics and Pharmacology Institute of Genetic Epidemiology Medical University of Innsbruck Innsbruck Austria; ^2^ Department of Vascular Surgery Medical University of Innsbruck Innsbruck Austria; ^3^ 3rd Medical Department of Metabolic Diseases and Nephrology Hietzing Hospital Vienna Austria; ^4^ Diabetes Research Group Faculty of Life Sciences and Medicine King's College London London UK; ^5^ Clinic of Angiology Center of Vascular Medicine Ernst von Bergmann Klinikum Potsdam Germany

**Keywords:** mitochondrial copy number, peripheral arterial disease, cardiovascular disease, mortality

## Abstract

**Background:**

Dysfunctional mitochondria have an influence on inflammation and increased oxidative stress due to an excessive production of reactive oxygen species. The mitochondrial DNA copy number (mtDNA‐CN) is a potential biomarker for mitochondrial dysfunction and has been associated with various diseases. However, results were partially contrasting which might have been caused by methodological difficulties to quantify mtDNA‐CN.

**Objective:**

We aimed to investigate whether mtDNA‐CN is associated with peripheral arterial disease (PAD) as well as all‐cause mortality and cardiovascular events during seven years of follow‐up.

**Methods:**

A total of 236 male patients with PAD from the Cardiovascular Disease in Intermittent Claudication (CAVASIC) study were compared with 249 age‐ and diabetes‐matched controls. MtDNA‐CN was measured with a well‐standardized plasmid‐normalized quantitative PCR‐based assay determining the ratio between mtDNA‐CN and nuclear DNA.

**Results:**

Individuals in the lowest quartile of mtDNA‐CN had a twofold increased risk for PAD which, however, was no longer significant after adjusting for leukocytes and platelets. About 67 of the 236 patients had already experienced a cardiovascular event at baseline and those in the lowest mtDNA‐CN quartile had a 2.34‐fold increased risk for these events (95% CI 1.08–5.13). During follow‐up, 37 PAD patients died and 66 patients experienced a cardiovascular event. Patients in the lowest mtDNA‐CN quartile had hazard ratios of 2.66 (95% CI 1.27–5.58) for all‐cause‐mortality and 1.82 (95% CI 1.02–3.27) for cardiovascular events compared with the combined quartile 2–4 (adjusted for age, smoking, CRP, diabetes, prevalent cardiovascular disease, leukocytes and platelets).

**Conclusion:**

This investigation supports the hypothesis of mitochondrial dysfunction in peripheral arterial disease and shows an association of low mtDNA‐CNs with all‐cause‐mortality and prevalent and incident cardiovascular disease in PAD patients with intermittent claudication.

## Graphical abstract

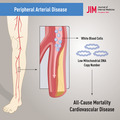

## Introduction

The muscles of patients with peripheral arterial disease (PAD) undergo several structural as well as metabolic changes. This results in reduced muscle function and strength which is mainly due to dysfunctional mitochondria and associated consequences. As mitochondria play an essential role in a number of mechanisms, their dysfunction leads to a multilevel failure and, in turn, causes a decline in muscle performance in patients suffering from vascular disease 1. Furthermore, dysfunctional mitochondria might be a key factor in the development of atherosclerosis via excessive production of reactive oxygen species (ROS) driving subclinical inflammation and increased oxidative stress 2. The mitochondrial DNA copy number (mtDNA‐CN) is a potential biomarker for mitochondrial dysfunction 3 and has been associated with various diseases 4.

Mitochondria as double‐membraned cell organelles supply energy by synthesizing adenosine triphosphate through oxidative phosphorylation 5. Each mitochondrion contains between 2 and 10 copies of the mitochondrial DNA, whereas up to 1000 mitochondria are present in each cell 6. An increased amount of mtDNA content has been associated with cancer risk 7 and metabolic disorders 8, whereas lower mtDNA‐CN is related to Parkinson’s disease 9 as well as the development and progression of cancer 10. Even though regulation mechanisms of mtDNA‐CN are widely unknown, it has been shown that mtDNA‐CN is specific to tissue types and developmental stage 11. The described associations are partially contrasting and might be influenced by the tissue in which mtDNA‐CN is measured and whether mtDNA is measured from cell‐free DNA or after extraction from cells.

The epidemiological evidence for an association between mtDNA‐CN measured in the peripheral blood and all‐cause mortality as well as prevalent and incident cardiovascular disease became only recently available. Large studies in the general population described low mtDNA‐CN to be significantly associated with all‐cause mortality 12 and various cardiovascular end‐points 13, 14, 15. Only few studies are available which investigated mtDNA‐CN in high‐risk patient groups such as patients with kidney impairment. We recently observed in almost 5000 patients with moderate kidney disease a strong association with all‐cause mortality as well as death due to infections 16. Other studies in patients with end‐stage kidney disease reported conflicting results 17, 18, 19. The studies on mtDNA‐CN and cancer are quite heterogeneous depending on cancer type and the source of cells investigated 20, 21.

Currently, few studies that have examined the role of mtDNA content in PAD led to conflicting results. Even though there are some indications of an influence of mitochondrial function on PAD, several questions remain unanswered 22, 23. Up to now, only one small study has investigated the association of mtDNA‐CN and clinical outcomes in PAD patients. This study measured the mtDNA‐CN in muscle biopsies 22. We hypothesized that a low mtDNA‐CN is a predictor for the presence of PAD as well as prospective outcomes such as all‐cause mortality and cardiovascular events in patients with PAD at baseline. We therefore performed an epidemiological approach with measurement of mtDNA‐CN in peripheral blood with a very well standardized and newly developed assay in patients with PAD and controls. This study was done in a case‐control design with a prospective follow‐up of all PAD patients over a period of seven years.

## Materials and methods

### Study population and outcome definition

The Cardiovascular Disease in Intermittent Claudication (CAVASIC) study is a case–control study with a prospective follow‐up investigation. Background and design of the study have been published earlier 24, 25 and more details are given in the Method S1. Briefly, 255 consecutive male patients with intermitted claudication (Fontaine Stage IIa or IIb regardless of whether they had already undergone a bypass surgery or intervention earlier) and 255 age‐ and diabetes‐matched controls were enrolled. Patient and controls were enrolled in two clinical centres, the Department of Vascular Surgery, Medical University Innsbruck and the 3rd Medical Department of Metabolic Diseases and Nephrology, Hietzing Hospital, Vienna, Austria. Exclusion criteria were presence of acute or critical limb ischaemia (Fontaine III or IV), impaired liver or kidney function, malignancy, past organ transplantation, and therapy with nicotinic acid or corticosteroids. Control individuals were from the same geographic region and matched with the patients with respect to age and presence of type 2 diabetes. We applied the same exclusion criteria to the control group as for patients. Control individuals with symptomatic PAD were excluded, but those with a history of cardiovascular disease were allowed to participate. Neither the patients nor the controls had acute illnesses or clinically detectable inflammatory processes at the time of enrolment. All study participants provided written informed consent, and the Ethical Committee of the participating study centres approved the examination protocol. All interviews and examinations were performed by one medical doctor at each of the two clinical centres and who was specially trained in vascular examinations and echocardiography to minimize interobserver bias. More details on the vascular examination procedure are provided in the Method S1. For this study, data on 236 patients and 249 controls were available for mitochondrial DNA copy number analysis (Figure S1).

In addition, outcome data of PAD patients were collected during a median follow‐up time of seven years. Clinical end‐points, such as fatal and nonfatal cardiovascular events and all‐cause mortality, were documented for all patients with intermittent claudication. The cardiovascular events were grouped into major adverse cardiovascular events (MACE) which included fatal cardiovascular events, nonfatal myocardial infarction and ischaemic cerebral infarction and an extended definition of cardiovascular events (CVD_ext_) which included – in addition to MACE – significant clinical events such as percutaneous transluminal coronary angioplasty (PTCA), aortocoronary bypass graft, angiographically proven coronary stenosis, transient ischaemic attack (TIA) and carotid endarterectomy.

### DNA extraction

The method of DNA extraction greatly influences the measurement of mtDNA‐CN as we demonstrated recently 26. Within the CAVASIC study, DNA was isolated from whole blood samples of patients and controls by a salting out method using the ‘Invitek Invisorb Blood Universal Kit’ (Stratec Molecular, Berlin, Germany).

### Plasmid‐normalized qPCR for mtDNA‐CN quantification

For quantification of mtDNA‐CNs, we applied a plasmid‐normalized quantitative real‐time polymerase chain reaction (qPCR)‐based assay that quantifies the mtDNA‐CN relative to the nuclear DNA as recently described 26. In contrast to other methods of mtDNA‐CN detection, this assay allows a reduction of inter‐assay variability thanks to the use of a dual insert plasmid containing a nuclear and a mitochondrial target. Details are provided in the Method S1. All samples were measured in triplicates and patients and controls were randomly distributed over the microplates to minimize plate‐specific or reader‐specific variations. Two control samples were measured in seven different experiments and revealed a coefficient of variation of 3.1% and 4.3% for the two samples, respectively. The laboratory personnel were not aware of the clinical history of patients and controls at the time of measurement.

### Statistical analysis

Baseline characteristics between PAD patients and controls were compared via descriptive statistics and presented as mean ± standard deviation (SD) and quartiles or N (%). Continuous variables were evaluated by using unpaired t‐tests or Wilcoxon rank‐sum test for non‐normally distributed variables, whereas categorical variables were analysed via χ^2^‐test. Logistic regression analyses were performed for different adjustment models to evaluate the association of mtDNA‐CN with PAD or prevalent cardiovascular disease in PAD patients. Kaplan–Meier curves were prepared for quartiles of mtDNA‐CN. Cox regression models for various adjustments were used to calculate hazard ratios (HRs) for overall mortality and incident cardiovascular events, including the corresponding 95% confidence intervals. Analyses for all‐cause mortality and incident cardiovascular events did not depart from the proportional hazard assumptions. Due to the limited number of clinical outcomes, we aimed for parsimonious models including only a restricted number of parameters. We mainly followed biological reasoning and only included variables with the potential of being a confounder (influence on both the outcome and mtDNA‐CN). For the logistic regression evaluating PAD risk, four different adjustment models were conducted: (i) adjusting for age; (ii) adjusting for age, hypertension, diabetes mellitus, HDL cholesterol; (iii) as model 2 plus additional adjustment for smoking; and (iv) as model 3 plus additional adjustment for leukocytes and platelets. The Cox regression models were adjusted for (i) age, (ii) age, current smoking, ln‐CRP, diabetes mellitus; (iii) as model 2 plus additional adjustment for prevalent CVD; and (iv) as model 3 plus adjustment for leukocytes and platelets. Further sensitivity analyses were conducted, where additional potential confounders were each added separately. mtDNA‐CN were modelled continuously and in quartiles. Moreover, a nonlinear p‐spline was created to evaluate the functional form of the relationship between mtDNA‐CN and risk of incident events. The results of the splines, the Kaplan–Meier curves, and the estimates for the quartiles 2, 3 and 4 compared with quartile 1 revealed that a categorical analysis of the data by combining the 2nd, 3rd and 4th quartile of mtDNA‐CN and to set quartile 1 as a reference fits the data better than modelling the data on a continuous scale. This procedure was also adopted to increase the informative value of the analyses and to simplify the interpretation of the generated data. All analyses were done using R 3.5.2. (Vienna, Austria, http://www.R-project.org).

## Results

### Association of mtDNA‐CN variation with PAD

The baseline characteristics of the 236 patients and 249 age‐ and diabetes‐matched controls available for analysis can be found in Table 1. There was no statistically significant difference in the mtDNA‐CN between PAD patients and controls (*P* = 0.29). However, the 25th and 50th percentiles for mtDNA‐CN were lower in patients than in controls, whereas the 75th percentile was higher, indicating a rather nonlinear relationship. The distribution of mtDNA‐CN for patients and controls is displayed in Figure S2. Due to the differences in the distribution, we performed a logistic regression analysis based on quartile categorization as presented in Table S1 applying different adjustments for confounders. Since the estimates for quartiles 2, 3 and 4 pointed in the same direction with no clear indication of a linear association, we combined quartiles 2–4 setting as the reference category to illustrate the influence of the lowest 25% values of mtDNA‐CN. This resulted in a 2.34‐fold increased risk for the first quartile compared with the combined quartiles 2–4 in an age‐adjusted model (OR = 2.34, 95% CI 1.53–3.06, *P* < 0.001). These findings remained stable when adjusted for further confounders in model 2 except when adjusted for the smoking status in model 3 (Table 2). As sensitivity analyses, all potential confounders were added separately to model 2. With the exception of leukocytes, no major changes in the odds ratios of mtDNA‐CN were observed (Table S2). The association between mtDNA‐CN and PAD was no longer significant after adjusting for platelets and especially for leukocytes (model 4 in Table 2). Spearman correlation analysis revealed that leukocyte count was inversely correlated with mtDNA‐CN in the entire group (*r* = −0.39), and this was slightly stronger in controls (*r* = −0.46) than in patients (*r* = −0.36) (all *P*‐values < 0.0001).

**Table 1:** Baseline characteristics of patients with peripheral arterial disease (PAD) and control individuals matched by age and diabetes mellitus

	Controls (*n *= 249)	PAD Patients (*n *= 236)	*P*‐value
Age (years)	56.9 ± 9.5 [50.0; 61.0; 64.0]	58.3 ± 6.3 [54.0; 59.5; 63.0]	0.96
Body Mass Index (kg m^−2^)	26.6 ± 3.9	26.7 ± 3.7	0.47
Current smoker, *n* (%)	30 (12.0%)	122 (52.4%)	<0.001
Diabetes Mellitus, *n* (%)	41 (16.5%)	35 (14.8%)	0.62
NT‐proBNP (pmol L^−1^)	9.2 ± 12.1 [4.2; 6.1; 9.7]	20.9 ± 35.1 [4.2; 10.9; 21.2]	<0.001
Total cholesterol (mg dL^−1^)	207.7 ± 35.1	205.7 ± 40.8	0.51
LDL cholesterol (mg dL^−1^)	135.8 ± 33.1	133.4 ± 37.1	0.35
HDL cholesterol (mg dL^−1^)	59 ± 16 [48; 57; 69]	49 ± 14 [41; 48; 54.25]	<0.001
Triglycerides (mg dL^−1^)	132 ± 80 [79; 111; 155]	172 ± 125 [95; 135; 211]	<0.001
C‐reactive protein (mg L^−1^)	2.6 ± 3.4 [1.0; 1.4; 2.8]	5.8 ± 6.4 [2.3; 4.2; 7.0]	<0.001
Leukocytes (G L^−1^)	5.8 ± 1.6 [4.5; 5.5; 6.7]	7.5 ± 2.1 [6.2; 7.3; 8.8]	<0.001
Platelets (G L^−1^)	224 ± 51 [190; 222; 256]	249 ± 60 [213; 243; 282]	<0.001
HbA_1c_ (%)	5.9 ± 0.9 [5.5; 5.6; 5.9]	6.0 ± 0.9 [5.5; 5.8; 6.1]	0.002
eGFR (mL min^−1^ 1.73m^−2^) a	81.7 ± 12.7	84.9 ± 15.0	<0.001
Serum Albumin (g L^−1^)	45.7 ± 3.9 [43.0; 45.6; 48.1]	44.6 ± 4.6 [41.6; 44.2; 47.1]	<0.001
Systolic blood pressure (mmHg)	140 ± 17	150 ± 20	<0.001
Diastolic blood pressure (mmHg)	82 ± 9	83 ± 10	0.33
Hypertension, *n* (%)^b^	148 (59.7%)	203 (86.0%)	<0.001
Cardiovascular disease, *n* (%)	19 (7.6%)	67 (28.4%)	<0.001
Ankle‐brachial index^c^	1.07 ± 0.12 [1.00; 1.08; 1.15]	0.72 ± 0.23 [0.54; 0.70; 0.89]	<0.001
Statin use, *n* (%)	36 (14.6%)	100 (43.1%)	<0.001
mtDNA copy number	113.6 ± 34.6 [90.4; 109.1; 132.2]	115.9 ± 50.3 [77.1; 102.4; 152.9]	0.29

Mean ± standard deviation (SD) [25th, 50th and 75th percentile in case of non‐normal distribution] or number (%).

a GFR denotes glomerular filtration rate calculated according to the CKD EPI equation 47.

b Hypertension was defined as systolic blood pressure ≥ 140 mmHg and/or diastolic blood pressure ≥ 90 mmHg, and/or receiving antihypertension treatment.

c The lowest ABI value from the 4 sites was used for data analysis. Individuals with ABI values >1.30 were excluded from this analysis.

**Table 2:** Logistic regression analysis investigating the association between mtDNA copy number and peripheral arterial disease at baseline in 236 patients and 249 controls. Results are given for quartile 1 versus quartiles 2–4 combined (=reference)

	mtDNA‐CN Quartile 1 vs. Quartile 2–4 (=reference)^a^	
	OR (95% CI)	*P*
Model 1 (adjusted for age)	2.34 (1.53–3.06)	<0.001
Model 2 (adjusted for age, hypertension, diabetes mellitus, HDL cholesterol)	2.45 (1.53–3.99)	<0.001
Model 3 (adjusted for age, hypertension, diabetes mellitus, HDL cholesterol and current smoking)	1.53 (0.90–2.62)	0.12
Model 4 (adjusted for age, hypertension, diabetes mellitus, HDL cholesterol, current smoking, leukocytes and platelets)	1.05 (0.57–1.93)	0.88

a Quartile 2–4 were merged and set as a reference. The odds ratios (OR) shown are calculated for quartile 1.

### Association of mtDNA‐CN with prevalent cardiovascular disease at baseline

At baseline, a higher proportion of patients with PAD had already experienced a CVD event when compared to controls (*n *= 67 or 28.4% vs. *n *= 19 or 7.6%, *P* < 0.001). We therefore investigated whether mtDNA‐CNs are associated with cardiovascular disease in the PAD patients at the time of the baseline investigation. In the fully adjusted model patients in the lowest quartile of mtDNA‐CN had a 2.34‐fold increased risk (95% CI 1.08–5.13, *P* = 0.03) for prevalent CVD (Table 3). The number of CVD cases was too small in the control group to allow a reliable analysis of the data.

**Table 3:** Logistic regression analysis investigating the association between mtDNA copy number and prevalent CVD (*n *= 67 events) in PAD patients (*n *= 236). Results are given for quartile 1 versus quartiles 2–4 combined (=reference)

	mtDNA‐CN Quartile 1 vs. Quartile 2–4 (=reference)^a^	
	OR (95% CI)	*P*‐value
Model 1 (adjusted for age)	1.44 (0.74–2.76)	0.28
Model 2 (adjusted for age, current smoking and HDL cholesterol)	1.95 (0.95–4.02)	0.07
Model 3 (adjusted for age, current smoking, HDL cholesterol and hypertension)	1.97 (0.95–4.10)	0.06
Model 4 (adjusted for age, current smoking, HDL cholesterol, hypertension, diabetes, leukocytes and platelets)	2.34 (1.08–5.13)	0.03

a Quartile 2–4 were merged and set as a reference. ORs are calculated for quartile 1.

### Association of mtDNA‐CN variation with mortality in PAD patients

**Figure 1:**
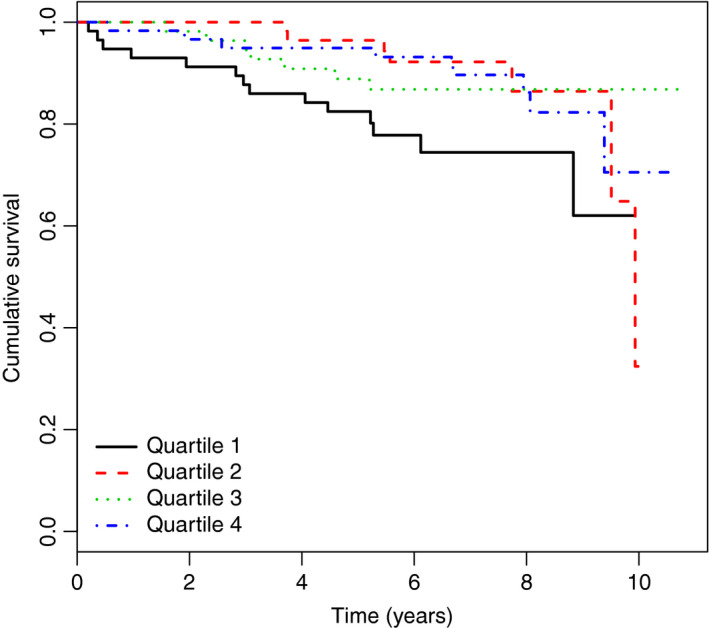
Kaplan–Maier survival curve for all‐cause mortality by quartiles of mtDNA copy number (*n *= 37). The x‐axis shows observation time in years, y‐axis represents the survival for each quartile of patients with 1 being 100%

**Figure 2:**
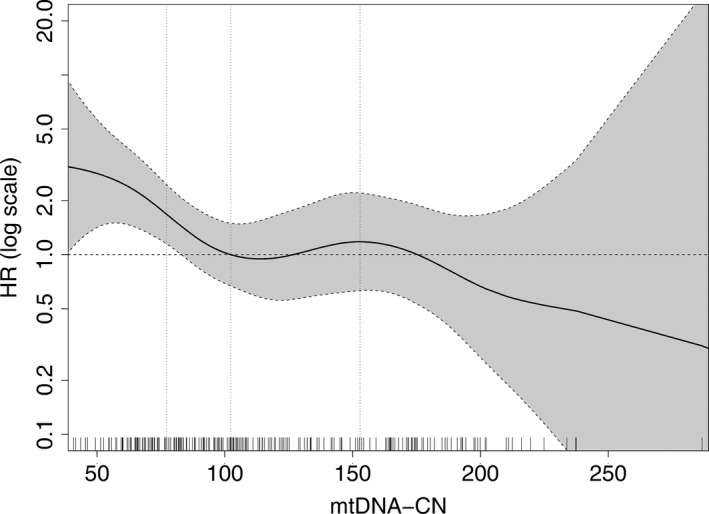
Adjusted nonlinear spline with 95% confidence bands demonstrating the association between mtDNA‐CN and all‐cause mortality (adjusted for age and smoking). On the y‐axis, the Hazard ratio (HR) is given as log‐scale and on the x‐axis, mitochondrial DNA copy number is shown. The median value of mtDNA copy number (102.36) is set as a reference with HR = 1 (horizontal dashed line). The vertical dashed lines show the 25th, 50th and 75th percentile of mtDNA‐CN. The rug plot at the bottom of the figures displays each single measured mtDNA‐CN value

During a median follow‐up of seven years, 37 of the 236 PAD patients (15.7%) died. Causes of death were cancer in 19 patients (51.4% of all deaths), vascular death in 12 patients (32.4%) and due to unknown causes in 6 patients (16.2%). A detailed characterization of patients’ mtDNA‐CN in quartiles, including the number of deaths, is provided in Table S3. A Kaplan–Meier survival curve for all‐cause mortality is shown in Fig. 1 (*P* = 0.05). Table S4 revealed similar hazard ratios for each of the quartiles 2, 3 and 4. This indicates an association of low mtDNA‐CN on an increased risk for all‐cause mortality in PAD patients in the first quartile of mtDNA‐CN when compared to quartiles 2–4. We observed 14 deaths in the lowest quartile and 7, 7, and 9 deaths in quartiles 2, 3 and 4, respectively. Furthermore, we created a p‐spline (adjusted for age and smoking status) as shown in Fig. 2. The results from the Kaplan–Meier survival curves, the estimates for each quartile and the spline analysis justified to combine quartiles 2–4 as reference group. Cox regression models applying different adjustments are shown in Table 4. Patients in the lowest quartile of mtDNA‐CN had a 3.27‐fold higher age‐adjusted risk of all‐cause mortality (HR = 3.27, 95% CI 1.64–6.50, *P* < 0.001). The results remained significant after adjustment for age, current smoking, CRP, prevalent CVD and diabetes (Model 3: HR = 2.62, 95% CI 1.27–5.40, *P* = 0.008). Even the additional adjustment for leukocytes and platelets did not change the estimates (model 4: HR = 2.66, 95% CI 1.27–5.58, *P* = 0.0098). In addition, the unexpected high number of cancer mortality was examined in detail. Patients in the lowest quartile of mtDNA copy numbers had a 3.43‐fold (95% CI 1.24–9.50, *P* = 0.018) higher risk for cancer mortality compared with those in quartiles 2–4 (data adjusted for age, smoking status, CRP, prevalent CVD and diabetes).

**Table 4:** Cox regression analysis investigating the association of mtDNA copy number and all‐cause mortality, major cardiovascular events and the extended definition of cardiovascular events during a median follow‐up of 7 years

	mtDNA‐CN Quartile 1 vs. Quartile 2–4 (=reference) a					
	All‐cause mortality (37 events)		Major cardiovascular events (35 events)		Extended definition of cardiovascular events (66 events)	
	HR (95% CI)	*P*	HR (95% CI)	*P*	HR (95% CI)	*P*
Model 1	3.27 (1.64–6.50)	<0.001	2.34 (1.14–4.79)	0.02	1.95 (1.14–3.33)	0.02
Model 2	2.79 (1.37–5.67)	0.005	2.10 (0.99–4.44)	0.05	1.81 (1.04–3.17)	0.04
Model 3	2.62 (1.27–5.40)	0.008	2.09 (0.98–4.46)	0.06	1.77 (1.01–3.92)	0.04
Model 4	2.66 (1.27–5.58)	0.0098	2.04 (0.92–4.50)	0.08	1.82 (1.02–3.27)	0.04

Model 1: adjusted for age; Model 2: adjusted for age, current smoking, ln‐CRP, diabetes mellitus; Model 3: adjusted for age, current smoking, ln‐CRP, diabetes mellitus, prevalent CVD; Model 4: adjusted for age, current smoking, ln‐CRP, diabetes mellitus, prevalent CVD, leukocytes and platelets.

^a^ Quartile 2–4 were merged and set as reference. The odds ratios (OR) displayed are calculated for quartile 1.

### Association of mtDNA‐CN variation with incident cardiovascular events

During follow‐up, we observed 35 major adverse cardiovascular events (MACE) and 66 events according to the extended definition (CVD_ext_). The hazard ratios for both outcome definitions were increased for the lowest quartile of mtDNA‐CN compared with the combined quartiles 2–4 (Table 4). The risk for MACE was 2.34‐fold increased for this quartile in the age‐adjusted model (95% CI 1.14–4.79, *P* = 0.02) and was only slightly weakened with further adjustment for potential confounders (fully adjusted model 4: HR = 2.04, 95% CI 0.92–4.50, *P* = 0.08). Similarly, the risk for the CVD_ext_ was 1.95‐fold increased for the lowest quartile (95% CI 1.14–3.33, *P* = 0.02) and remained significant for all adjustment models (fully adjusted model 4: HR = 1.82, 95% CI 1.02–3.27, *P* = 0.04).

## Discussion

### Main findings

The present study contains two components: a case–control study of patients with PAD and controls without PAD and a prospective observation period of 7 years for the PAD patients. In the case–control design, we found low mtDNA‐CN to be associated not only with the risk for peripheral arterial disease but also with prevalent CVD at baseline. The association with PAD, however, vanished as soon as we adjusted for leukocyte count. During the prospective follow‐up, we found the lowest quartile of mitochondrial DNA copy number to be significantly associated with a 2.66‐fold increased risk for all‐cause mortality. Furthermore, the risk for incident cardiovascular events during follow‐up was roughly doubled for those PAD patients in the lowest quartile of mtDNA‐CN compared with the combined quartiles 2–4.

### mtDNA‐CN and PAD

To the best of our knowledge, this is the first study which investigated mtDNA‐CN in the peripheral blood of PAD patients. We found that a lower mtDNA‐CN was associated with an increased risk for PAD. The only other available study evaluating the role of mtDNA in PAD patients performed mtDNA‐CN measurements not in peripheral blood but in calf muscle biopsy material of 34 PAD patients and 10 controls. They found a higher mtDNA‐CN to be associated with a lower ankle‐brachial index 22. These results are contrasting at first glance but might be explained by the site of mtDNA‐CN measurement. McDermott et al. studied local effects directly in the muscles 22 and not systemic effects in the peripheral blood stream as we did. It is conceivable that a higher number of mitochondria might have been produced locally in the calf muscle to ensure the energy supply necessary in the weakened muscles resulting in a site‐specific increase in mtDNA‐CN 1, 11.

It was interesting to observe that the association between mtDNA‐CN and PAD vanished as soon as we adjusted the data for the leukocyte count. It is not completely clear what comes first, the disturbance in mtDNA homeostasis followed by atherosclerosis or vice versa. Leukocytes were significantly higher in cases than controls and might reflect inflammatory processes. Therefore, the prediction of the case versus control status might be rather related to the discriminatory ability of the investigated variables than the pathogenetic mechanisms. This would mean that adjusting for leukocyte count might result in an overadjustment of the data since a high leukocyte count might be rather a consequence than a cause of PAD. The prospective data analysis might suggest that the mtDNA‐CN is already low at baseline and predicts the development of clinical end‐points in the future.

At first sight, the association between mtDNA‐CN and PAD in our study seemed to be confounded by smoking. However, the interpretation and generalizability of this finding has to be taken with caution especially due to the low number of only 30 smokers in the control group and subsequent uneven distribution in the various quartiles of mtDNA with no discernable pattern. The logistic regression model building might have become quite instable by only nine smokers in quartile 1 of the controls. However, other data from our group indicate that smoking does not fulfil the prerequisite of a confounder: we could not observe any association between smoking and mtDNA‐CN in the German Chronic Kidney Disease study with almost 5000 participants 16. In contrast to the baseline analysis between PAD cases and controls, the association between mtDNA‐CN and outcomes observed during the prospective observation period were not markedly influenced by smoking.

### All‐cause mortality

We observed a strong association between low mtDNA‐CN and all‐cause mortality in PAD patients. Similar but less strong associations have been observed in the general population as well as in patients with impaired kidney function 12, 16. For example, the general population sample from the Cardiovascular Health Study (CHS) and the Atherosclerosis Risk in Communities Study (ARIC) including 16 401 individuals showed that participants in the lowest compared with the highest quintile of mtDNA‐CN had 1.33‐fold increased risk to die after extended adjustment for common risk factors 12. The results from 4812 patients with impaired kidney function are from our laboratory using the same method as in the present study. Patients in the lowest quartile of copy numbers had a 1.81‐fold increased risk for all‐cause mortality after adjusting for kidney function and cardiovascular risk factors 16. Small studies in patients with end‐stage kidney disease reported conflicting results 17, 18, 19.

We see the additional analyses we performed for death due to cancer with caution due to the limited number of 19 cases. However, the risk was more than threefold increased in the lowest quartile of mtDNA‐CN. Currently, contrary information on the association between mtDNA‐CN and cancer can be found in the literature reporting decreased as well as increased mtDNA‐CN in patients with cancer 20, 21. One of the reasons for these diverging results might be whether these measurements are done in the tumour tissue, peripheral blood mononuclear cells or whole blood or from cell‐free circulating mtDNA. Recent data revealed that tumour cells of many types of cancer have fewer copies of mitochondrial DNA than the cells that make up normal tissue 27. On the other hand, mtDNA copies can be released into the circulation from damaged or disintegrating tumour cells and might result in a preponderance of mtDNA‐CN when these copies are measured only in cell‐free DNA 20. As we discussed recently 16, the measurement of mtDNA copy numbers from nuclear cells as done in our study is an attempt to assess relative mitochondrial abundance within cells, and therefore a higher mtDNA copy number is expected to be associated with beneficial outcomes as observed in the present study.

### Cardiovascular disease

Patients with PAD have an elevated risk for prevalent and incident cardiovascular disease 28, 29, 30. We investigated in the present study whether mtDNA‐CN is associated with this often life‐limiting end‐point. We found a roughly doubling of risk for prevalent and incident CVD events for those PAD patients in the lowest quartile of mtDNA‐CN which was independent from major PAD and CVD risk factors. The data of this high‐risk group are in line with observations from the general population which also showed significant associations in case–control studies 31, 32, 33 as well as prospective studies for various CVD end‐points 13, 14, 15. For example a study including three prospective, population‐based cohort studies with 21 870 participants found lower mtDNA‐CN to be independently associated with prevalent as well as incident cardiovascular disease 13.

### Possible explanations and clinical implications of the findings

Mitochondrial dysfunction disrupts cell homeostasis and has been hypothesized to be involved in the development and progression of various chronic diseases including atherosclerosis. However, it is still a matter of discussion how dysfunctional mitochondria contribute to this pathological condition. Animal studies have shown that mtDNA damage occurs before arterial walls lesions 34, and in an atherosclerotic mouse model mtDNA defects and reduced respiratory activity have been found 35. Furthermore, human plaque samples displayed decreased mtDNA content and impaired respiration 36. These findings suggest a link between mtDNA damage and atherosclerosis but the precise mechanism underlying this relationship and the exact role of mitochondrial ROS are still poorly understood. Inflammation and an increased oxidative stress due to an excessive production of ROS are key drivers in the development of atherosclerosis 2. ROS is able to oxidize lipids, promotes the uptake of inflammatory cells into the vessel wall and enhances the proliferation of vascular smooth muscle cells 37. Dysfunctional mitochondria result in a release of mtDNA into the circulation which might induce an inflammatory state 38, 39. This indicates ROS as a pro‐atherogenic factor, but other studies suggest a mitochondrial contribution to atherosclerosis independent from ROS production. MtDNA dysfunction can result in disturbances in the electron transport chain with a reduced ATP content in the vascular smooth muscle cells. This can promote apoptosis and inhibit the cell proliferation resulting in atherosclerosis and plaque rupture 34, 40. Monocyte cell death and an increased release of inflammatory cytokines can follow 41.

The clinical implications of the findings are as follows: on the one hand, the measurement of mtDNA‐CN might improve risk prediction especially for the patients with very low mtDNA‐CN. On the other hand, mitochondria‐targeted therapies are currently under investigation and aim to decrease the arterial stiffening with ageing which might be a hallmark of many age‐related diseases. For example preclinical studies in mice receiving mitochondria‐targeted antioxidant MitoQ resulted in a decrease of aortic stiffness of older mice by attenuation and reversal of age‐related aortic elastin degradation 42. A randomized placebo‐controlled, double‐blind cross‐over trial with MitoQ in healthy older adults with impaired endothelial function resulted in an improvement of vascular endothelial function, a reduction in aortic stiffness and a decrease in oxidized LDL. This was most likely caused by a suppression of mitochondrial ROS production 43. Mitochondrial dysfunction with ROS production seems to play also a major role in the development of intestinal ischaemia reperfusion often observed in haemorrhagic shock, trauma, sepsis, burns and surgical procedures 44, 45. This disruption of the mucosal barrier opens the route for entrance of bacteria and their antigens resulting in severe infections as well as the amplification of an inflammatory response. It has been shown that intestinal ischaemia reperfusion reduced the mtDNA‐CN and the mRNA transcription level inducing mitochondrial disruption in the intestinal epithelial cells. In an experimental model, a pretreatment with MitoQ inhibited these deleterious effects 46. This and similar therapies are still in the experimental phase but might open new avenues for therapies of common ageing‐related diseases.

### Strengths and limitations of the study

This study is not only based on a case–control design with a baseline investigation but also a prospective follow‐up. We used a very thoroughly standardized assay for mtDNA‐CN measurement using a plasmid normalization approach which we recently demonstrated to have a very pronounced and positive effect on the coefficient of variation of the measured values 26. Furthermore, we used the same DNA extraction method throughout the study in patients and controls. This is of crucial importance for the measured values since we demonstrated recently that the DNA extraction method has a huge impact on measurement of mtDNA‐CN 26. Furthermore, we handled the samples from patients and controls in the same way and arranged the samples from patients and controls alternately on each plate to avoid any batch effects.

Limitations of the study include the restricted number of individuals and clinical end‐points. However, we calculated that we would be able to detect at least an OR of 1.8 in the logistic regression analysis given a power of 80% and an HR of 2.2 for the CVD events and 2.9 for all‐cause mortality in the Cox analysis. The observed estimates were even higher. Furthermore, we performed a careful statistical model building based on biological and epidemiological principles to avoid the inclusion of too many variables in the regression models considering the number of events observed. With the building of different models step by step, we tested the stability of the observed association between mtDNA‐CN and various outcomes. Other limitations were the restriction to Caucasian ethnicity and male patients and controls which has been decided at the baseline of the study to reach better homogeneity of the study population.

## Conclusions

This study showed an association between reduced mtDNA‐CN and all‐cause mortality in male PAD patients. We furthermore observed an association between the lowest quartile of mtDNA‐CN and cardiovascular disease at baseline as well as for incident cardiovascular events during the seven years of follow‐up. Further studies are required to understand and characterize the mechanisms underlying this association.

## Conflict of interest

The authors declare no competing interests.

## Supporting information


**Method S1.** Additional information on the CAVASIC study as well as further details on mtDNA copy number quantification via plasmid‐normalized qPCR.
**Table S1.** Logistic regression analysis investigating the association between mtDNA copy number and peripheral arterial disease at baseline in 236 patients and 249 controls.
**Table S2.** Additional sensitivity analyses for the logistic regression analysis presented in Table 2 of the main manuscript.
**Table S3.** Baseline characteristics of patients with peripheral arterial disease analyzed by quartiles. Dataset was split based on mtDNA copy number of patients only.
**Table S4.** Cox regression analysis investigating the association of mtDNA copy number and all‐cause mortality, MACE and CVDext (median follow‐up 7 years).
**Figure S1.** Flow chart illustrating the study design of the CAVASIC Study and demonstrating the number of cases included in the final analyses.
**Figure S2.** Distribution of mtDNA copy number (x‐axis) shown for patients with peripheral arterial disease and controls.
