# Supplementary material for: Mitochondrial DNA copy number is associated with all‐cause mortality and cardiovascular events in patients with peripheral arterial disease
Source: J Intern Med. 2020 Feb 9;287(5):569–79. doi: 10.1111/joim.13027 (PMC7318579; doi:10.1111/joim.13027)
Supplement: Supplementary file 1 — Method S1. Additional information on the CAVASIC study as well as further details on mtDNA copy number quantification via plasmid‐normalized qPCR. Table S1. Logistic regression analysis investigating the association between mtDNA copy number and peripheral arterial disease at baseline in 236 patients and 249 controls. Table S2. Additional sensitivity analyses for the logistic regression analysis presented in Table 2 of the main manuscript. Table S3. Baseline characteristics of patients with peripheral arterial disease analyzed by quartiles. Dataset was split based on mtDNA copy number of patients only. Table S4. Cox regression analysis investigating the association of mtDNA copy number and all‐cause mortality, MACE and CVDext (median follow‐up 7 years). Figure S1. Flow chart illustrating the study design of the CAVASIC Study and demonstrating the number of cases included in the final analyses. Figure S2. Distribution of mtDNA copy number (x‐axis) shown for patients with peripheral arterial disease and controls. [file JOIM-287-569-s001.pdf]

Supplemental Material for

Mitochondrial DNA copy number is associated with all-cause mortality and cardiovascular events in patients with peripheral arterial disease

Adriana Koller, BSc [1], Federica Fazzini, PhD [1], Claudia Lamina, PhD [1], Barbara Rantner, PhD [2], Barbara Kollerits, MD [1], Marietta Stadler, MD [3,4], Peter Klein-Weigel, MD [5], Gustav Fraedrich, MD [2], Florian Kronenberg, MD [1]

[1] Institute of Genetic Epidemiology, Department of Genetics and Pharmacology, Medical University of Innsbruck, Innsbruck, Austria

[2] Department of Vascular Surgery, Medical University of Innsbruck, Innsbruck, Austria

[3] 3rd Medical Department of Metabolic Diseases and Nephrology, Hietzing Hospital, Vienna, Austria

[4] Diabetes Research Group, Faculty of Life Sciences and Medicine, King's College London, London, United Kingdom

[5] Clinic of Angiology, Center of Vascular Medicine, Ernst von Bergmann Klinikum, Potsdam, Germany

Background to the CAVASIC study

The following Supplemental Material lists main parts from the earlier description of the CAVASIC Study [(1)] which are currently not yet described in the main part of the manuscript.

The CAVASIC Study (**CA**rdio**VAS**cular disease in **I**ntermittent **C**laudication) is a prospective case-control study which was initiated in 2002 to identify cardiovascular risk factors in patients with intermittent claudication.

For the measurement of the ankle-brachial index (ABI) the systolic brachial blood pressure was initially measured once on both arms and two further measurements were done on the arm with the higher systolic value. The mean value of these two additional measurements on the arm with the higher systolic value was used for further calculations. Measurements of the systolic blood pressures on the lower extremity were done three times for each artery (arteria dorsalis pedis and arteria tibialis posterior, each on the left and right ankle). The mean value of the second and third measurement for each site was used for the ABI calculation for each of the four sites. The ABI was calculated as the ratio of the systolic blood pressures (=mean value of the second and third measurement) of each of the four sites of the lower extremity to the mean systolic blood pressure of the arm. For data analysis the lowest ABI value from the four sites was used for further data analysis which results in a higher sensitivity of the ABI [(2)] and which is in line with the "REduction of Atherothrombosis for Continued Health" (REACH) Registry [(3)].

The Edinburgh questionnaire was used to identify symptomatic intermittent claudication [(4)]. Patients were considered as having PAD if they presented with symptoms of an intermittent claudication and an ABI below 0.90. Furthermore, we performed an oszillography and did a standardized constant load treadmill examination (12 percent acceleration and 3.0 km per hour) to evaluate the walking distance. If any further therapy was planned, additional ultrasound scanning or magnetic resonance imaging of the arteries of the lower extremity was done. In case of further endovascular treatment, a conventional angiography was carried out.

Demographic data, clinical history, smoking, alcohol consumption, diet, amount of leisure and working time physical activity as well as atherosclerosis risk profile were recorded by a standardized interview. Medications at the baseline exam were recorded. All participants underwent a clinical examination with cardiological and angiological focus including an electrocardiography and an echocardiography.

Participants were diagnosed having diabetes mellitus if the fasting plasma glucose was >126mg/dL and/or if they were treated with antidiabetic drugs. They were considered as hypertensive when the systolic blood pressure was ≥140mm Hg and/or the diastolic blood pressure was ≥90mm Hg or if they were treated with antihypertensive drugs.

EDTA, citrate plasma and serum samples were obtained after a 10-14h overnight fasting period and were immediately processed. Samples were stored in aliquots at -80°C until laboratory measurements.

mtDNA copy number quantification via plasmid-normalized qPCR

In order to verify whether the two fluorescent dyes present different intensities of fluorescence emission and to normalize for differences between runs (master mix performance, instrument calibration, environmental variability, etc.), a linearized dual insert plasmid was created, containing the mtDNA insert (108 bp) and the nuclear insert (86 bp).

We used the same qPCR protocol as described previously [(5)]. In brief, the analysis has been performed on the QuantStudio™ 6 Flex system instrument (Thermo Fisher Scientific, Waltham, MA, USA) and samples, as well as positive and negative controls were measured in triplicates. The plasmid was used in two different dilutions (diluted with TE) and measured in quadruplets. For mtDNA, the reporter was FAM, whereas for nuclear DNA samples, Yamika Yellow (an alternative to VIC) was utilized as a dye. The qPCR was performed using the following conditions: 95 °C for 3 minutes for initial polymerase activation, 40 cycles of 95 °C for 15 sec and 60 °C for 1 min. PCR amplification was run in a 10 µl reaction consisting of: 5 µl Brilliant III Ultra-Fast qPCR Master Mix with low ROX (Agilent Technologies, Santa Clara, CA, USA), 1 µl mtDNA primers (300 nM each), 1 µl nDNA primers (600 nM each), 1 µl mtDNA probe (300 nM), 1 µl nDNA probe (300 nM) and 1 µl DNA (3–5 ng).

The mtDNA copy number was calculated using the Delta Delta Cq (quantification cycle) method: $2 \times E^{-(\Delta Cq\ sample - \Delta Cq\ plasmid)}$, where "E" is the average mean efficiency of the PCR of the PCR reaction of the two targets [(6)] and "2" is the account for the two copies of nuclear DNA in a cell. The primer and probe sequences were modified from Bai et al. [(7)] and synthesized by Microsynth AG (Balgach, Switzerland). In brief, a region of mt-tRNA^{Leu} was amplified using the forward primer 5'- CACCCAAGAACAGGGTTTGT and the reverse primer 5'- TGGCCATGGGTATGTTGTTA; a region of beta-2-microglobulin was amplified using the forward primer 5'- TGCTGTCTCCATGTTTGATGTATCT and the reverse primer 5'- TCTCTGCTCCCCACCTCTAAGT. Probe sequences were FAM - 5'- TTAACCGGGCTCTGCCATCT for mt-tRNA^{Leu} and Yamika Yellow - 5'- CAGGTTGCTCCACAGGTAGCTCTAG for beta-2-microglobulin.

Supplemental Table 1: Logistic regression analysis investigating the association between mtDNA copy number and peripheral arterial disease at baseline in 236 patients and 249 controls. Results are given for each quartile and quartile 1 was used as reference.

		mtDNA-CN in quartiles (Quartile 1 = reference)	
		OR (95%CI)	P
**Model 1** (adjusted for age)			
	2nd Quartile	0.45 (0.26-0.75)	0.002
	3rd Quartile	0.22 (0.12-0.37)	<0.001
	4th Quartile	0.76 (0.45-1.28)	0.30
**Model 2** (adjusted for age, HDL Cholesterol, hypertension and diabetes)			
	2nd Quartile	0.44 (0.25-0.78)	0.005
	3rd Quartile	0.20 (0.11-0.36)	<0.001
	4th Quartile	0.74 (0.40-1.34)	0.33
**Model 3** (adjusted for age, hypertension, diabetes, HDL cholesterol and current smoking)			
	2nd Quartile	0.79 (0.40-1.46)	0.43
	3rd Quartile	0.28 (0.14-0.56)	<0.001
	4th Quartile	1.21 (0.63-2.35)	0.56

Supplemental Table 2: Additional sensitivity analyses for the logistic regression analysis presented in Table 2 of the main manuscript. This investigates the association between mtDNA copy number and peripheral arterial disease at baseline in 236 patients and 249 controls. Results are given for quartile 1 versus quartiles 2-4 combined (=reference).

	**mtDNA CN Quartile 1 vs. Quartile 2-4 (=reference)** *	
	OR (95%CI)	P
**Model 2** (adjusted for age, hypertension, diabetes mellitus, HDL cholesterol) as described in Table 2 of the main manuscript	2.45 (1.53-3.99)	<0.001
**Additional sensitivity analyses**		
as model 2 plus NT-proBNP	2.24 (1.38-3.67)	<0.001
as model 2 plus ln-CRP	1.93 (1.18-3.18)	0.009
as model 2 plus HbA1c	2.54 (1.58-4.16)	<0.001
as model 2 plus eGFR	2.50 (1.54-4.12)	<0.001
as model 2 plus serum albumin	2.35 (1.46-3.82)	<0.001
as model 2 plus triglycerides	2.46 (1.53-4.01)	<0.001
as model 2 plus cardiovascular disease	2.43 (1.50-3.99)	<0.001
as model 2 plus leukocytes	1.11 (0.64-1.91)	0.72
as model 2 plus platelets	2.24 (1.43-4.07)	<0.001

* Quartile 2-4 were merged and set as a reference. The odds ratios (OR) shown are calculated for quartile 1.

Supplemental Table 3: Baseline characteristics of patients with peripheral arterial disease analyzed by quartiles. Dataset was split based on mtDNA copy number of patients only. Quantitative data are given as mean [25th, 50th and 75th percentile]

mtDNA CN range	**Q1** (n=59) 38.2-76.8	**Q2** (n=59) 76.9-102.3	**Q3** (n=59) 102.4-152.7	**Q4** (n=59) 152.8-312.3	P-value
Age (years)	57.2 [53.5; 58.0; 62.0]	59.1 [54.5; 60.0; 64.0]	58.2 [53.0; 58.0; 64.0]	58.6 [55.5; 61.0; 63.0]	0.32
Body Mass Index (kg/m²)	26.67	27.07	26.93	26.03	0.62
Current smokers, n (%)	45 (76.3%)	21 (37.5%)	31 (52.5%)	25 (42.4%)	<0.001
Diabetes Mellitus, n (%)	9 (15.3%)	13 (22.0%)	11 (18.6%)	2 (3.4%)	0.027
NT-proBNP (pmol/L)	26.4 [6.1; 11.6; 19.5]	14.1 [4.2; 10.1; 16.3]	26.9 [6.3; 11.6; 29.7]	16.7 [5.2; 10.6; 20.8]	0.36
Total cholesterol (mg/dL)	208.46	202.46	206.59	205.32	0.89
LDL cholesterol (mg/dL)	136	130.3	133.1	134.1	0.91
HDL cholesterol (mg/dL)	52 [42; 48; 54]	51 [44; 50; 57]	47 [40; 47; 52]	47 [38; 46; 53]	0.14
Triglycerides (mg/dL)	164 [102; 140; 192]	182 [103; 132; 224]	181 [91; 131; 250]	163 [92; 139; 203]	0.86
C-reactive protein (mg/L)	7.32 [3.00; 5.40; 10.05]	5.38 [1.65; 3.40; 6.00]	4.98 [2.95; 4.30; 6.35]	5.64 [1.70; 2.80; 6.60]	0.005
HbA_{1c} (%)	5.95 [5.45; 5.70; 6.19]	6.22 [5.70; 5.90; 6.20]	6.17 [5.63; 5.90; 6.20]	5.76 [5.50; 5.70; 6.00]	0.032
eGFR (mL/min/1.73m²)	86.9 [76.1; 90.6; 97.9]	84.2 [76.7; 85.7; 92.9]	86.1 [74.1; 88.3; 98.7]	82.4 (73.3; 81.0; 92.7]	0.17
Albumin (g/L)	44.0 [41.1; 44.0; 46.5]	45.3 [42.6; 45.4; 48.0]	44.1 [41.6; 43.9; 46.6]	44.9 [41.4; 43.9; 47.9]	0.29
Leukocytes (G/L)	8.8 [7.3; 8.4; 9.8]	7.4 [6.0; 7.1; 8.8]	7.3 [6.2; 7.2; 8.5]	6.7 [5.9; 6.6; 7.7]	<0.001
Platelets (G/L)	257 [223; 250; 274]	227 [196; 225; 255]	252 [225; 254; 295]	258 [216; 254; 291]	0.005
Systolic blood pressure (mmHg)	148 [133; 145; 165]	152 [139; 150; 164]	152 [140; 150; 160]	150 [135; 150; 164]	0.75
Diastolic blood pressure (mmHg)	81 [79; 80; 90]	84 [80; 80; 90]	84 [80; 80; 90]	84 [80; 80; 90]	0.48
Hypertension, n (%)	49 (83%)	52 (88%)	52 (88%)	50 (85%)	0.81
Cardiovascular disease, n (%)	19 (32%)	13 (22%)	20 (34%)	15 (25%)	0.44
Ankle-brachial index	0.67 [0.51; 0.62; 0.87]	0.76 [0.58; 0.70; 0.86]	0.70 [0.50; 0.71; 0.88]	0.77 [0.60; 0.78; 0.93]	0.062
Statin use, n (%)	21 (35.6%)	23 (41.8%)	28 (47.5%)	28 (47.5%)	0.51
Total mortality	14 (23.7%)	7 (12.1%)	7 (11.8%)	9 (15.3%)	0.25
Cancer mortality	8 (13.6%)	4 (6.8%)	3 (5.1%)	4 (6.8%)	0.34
Vascular death	4 (6.8%)	1 (1.7%)	4 (6.8%)	3 (5.1%)	0.56

Supplemental Table 4: Cox regression analysis investigating the association of mtDNA copy number and all-cause mortality, MACE and CVD_{ext} (median follow-up 7 years). Results are given for each quartile and quartile 1 was used as reference.

	mtDNA-CN in quartiles (Quartile 1 = reference)					
	**All-cause mortality (37 events)**		**Major cardiovascular events (35 events)**		**Extended definition of cardiovascular events (66 events)**	
	HR (95%CI)	P	HR (95%CI)	P	HR (95%CI)	P
**Model 1**						
2nd Quartile	0.29 (0.12-0.73)	0.009	0.23 (0.07-0.72)	0.01	0.41 (0.20-0.86)	0.02
3rd Quartile	0.29 (0.12-0.74)	0.01	0.75 (0.33-1.69)	0.48	0.69 (0.36-1.31)	0.25
4th Quartile	0.33 (0.14-0.78)	0.01	0.34 (0.13-0.88)	0.03	0.45 (0.23-0.90)	0.02
**Model 2**						
2nd Quartile	0.30 (0.11-0.81)	0.02	0.30 (0.09-0.95)	0.04	0.41 (0.19-0.87)	0.02
3rd Quartile	0.36 (0.14-0.91)	0.03	0.82 (0.36-1.89)	0.64	0.77 (0.40-1.47)	0.42
4th Quartile	0.42 (0.17-1.01)	0.05	0.32 (0.11-0.90)	0.03	0.49 (0.24-1.01)	0.05
**Model 3**						
2nd Quartile	0.33 (0.12-0.90)	0.03	0.29 (0.09-0.95)	0.04	0.43 (0.20-0.92)	0.03
3rd Quartile	0.37 (0.14-0.93)	0.04	0.82 (0.35-1.89)	0.63	0.77 (0.40-1.47)	0.43
4th Quartile	0.45 (0.18-1.09)	0.08	0.32 (0.11-0.89)	0.03	0.50 (0.24-1.03)	0.06
**Model 4**						
2nd Quartile	0.27 (0.08-0.88)	0.03	0.28 (0.07-1.09)	0.07	0.47 (0.21-1.05)	0.07
3rd Quartile	0.38 (0.15-0.98)	0.04	0.81 (0.34-1.94)	0.64	0.70 (0.36-1.38)	0.31
4th Quartile	0.45 (0.18-1.15)	0.10	0.33 (0.11-0.97)	0.04	0.46 (0.22-0.98)	0.045

Model 1: adjusted for age
Model 2: adjusted for age, current smoking, ln-CRP, diabetes
Model 3: adjusted for age, current smoking, ln-CRP, diabetes, prevalent CVD
Model 4: adjusted for age, current smoking, ln-CRP, diabetes mellitus, prevalent CVD, leukocytes and platelets

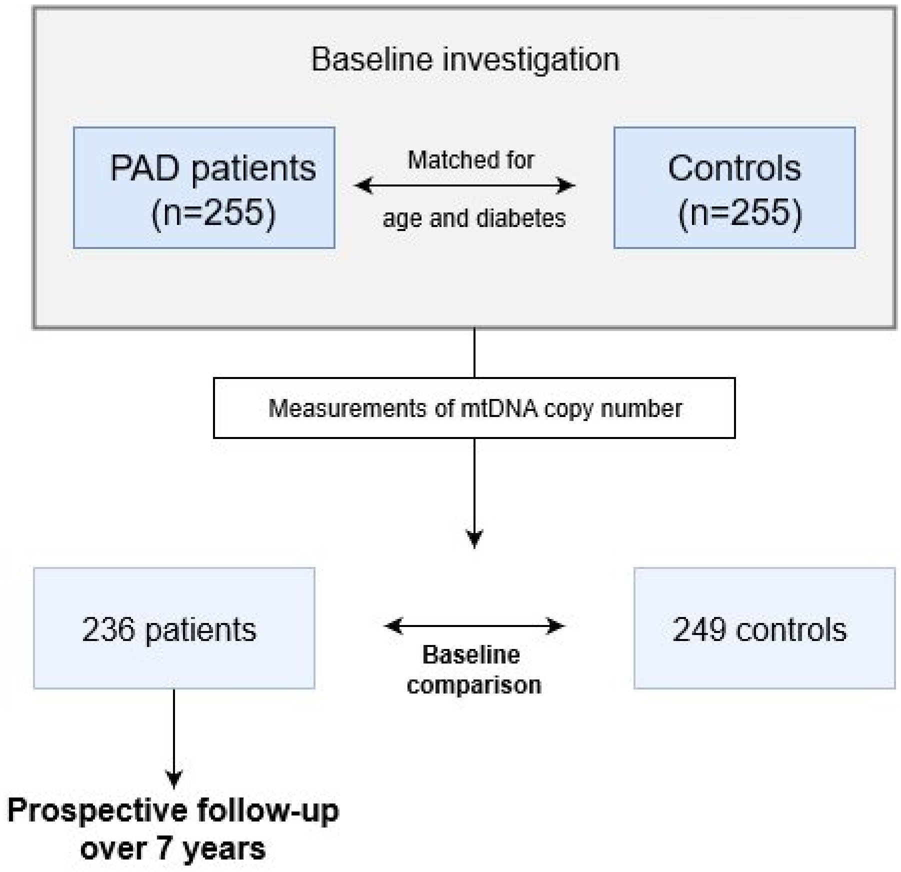

Supplemental Figure 1: Flow chart illustrating the study design of the CAVASIC Study and demonstrating the number of cases included in the final analyses.

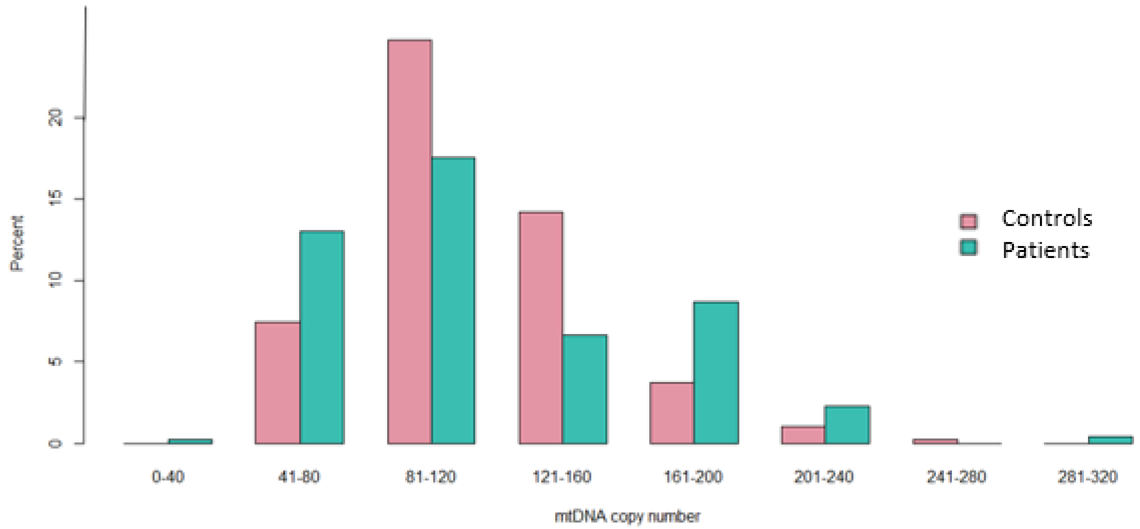

Supplemental Figure 2: Distribution of mtDNA copy number (x-axis) shown for patients with peripheral arterial disease and controls. The frequency is given in percent on the y-axis.

References

1. Rantner B, Kollerits B, Anderwald-Stadler M, Klein-Weigel P, Gruber I, Gehringer A, Haak M, Schnapka-Köpf M, Fraedrich G, Kronenberg F: Association between the *UGT1A1* TA-repeat polymorphism and bilirubin concentration in patients with intermittent claudication: results from the CAVASIC Study. ***Clin. Chem.*** 54:851-857, 2008.
2. Schröder F, Diehm N, Kareem S, Ames M, Pira A, Zwettler U, Lawall H, Diehm C: A modified calculation of ankle-brachial pressure index is far more sensitive in the detection of peripheral arterial disease. ***J Vasc. Surg.*** 44:531-536, 2006.
3. Ohman EM, Bhatt DL, Steg PG, Goto S, Hirsch AT, Liau CS, Mas JL, Richard AJ, Rother J, Wilson PW: The REduction of Atherothrombosis for Continued Health (REACH) Registry: an international, prospective, observational investigation in subjects at risk for atherothrombotic events-study design. ***Am Heart J*** 151:786-10, 2006.
4. Leng GC, Fowkes FG: The Edinburgh Claudication Questionnaire: an improved version of the WHO/Rose Questionnaire for use in epidemiological surveys. ***J. Clin. Epidemiol.*** 45:1101-1109, 1992.
5. Fazzini F, Schopf B, Blatzer M, Coassin S, Hicks AA, Kronenberg F, Fendt L: Plasmid-normalized quantification of relative mitochondrial DNA copy number. ***Sci. Rep.*** 8:15347, 2018.
6. Pfaffl MW: A new mathematical model for relative quantification in real-time RT-PCR. ***Nucleic Acids Res.*** 29:e45, 2001.
7. Bai RK, Wong LJ: Simultaneous detection and quantification of mitochondrial DNA deletion(s), depletion, and over-replication in patients with mitochondrial disease. ***J. Mol. Diagn.*** 7:613-622, 2005.
